# Influence of Build Orientation and Part Thickness on Tensile Properties of Polyamide 12 Parts Manufactured by Selective Laser Sintering

**DOI:** 10.3390/polym16162241

**Published:** 2024-08-07

**Authors:** Jonathan J. Slager, Brian C. Earp, Ahmed M. Ibrahim

**Affiliations:** 1 Department of Mechanical and Nuclear Engineering, United States Naval Academy, Annapolis, MD 21401, USA; earp@usna.edu; 2 Department of Naval Architecture and Ocean Engineering, United States Naval Academy, Annapolis, MD 21401, USA; ibrahim@usna.edu

**Keywords:** additive manufacturing (AM), selective laser sintering (SLS), polyamide-12 (PA12), orthotropic, orientation and thickness affects, modeling

## Abstract

The use of additive manufacturing to rapidly test and evaluate solutions to engineering problems has been demonstrated. Selective laser sintering (SLS) is a subset of additive manufacturing that is particularly well suited to producing structural thin wall models and end use parts which can improve the ability to prototype and manufacture certain designs at a substantially lower cost and time compared to current methods. However, a more comprehensive understanding of the material properties of these parts is warranted. The presented research investigates the influence of print orientation and sample thickness on the material properties of printed SLS parts. This novel work involves holding a hatch pattern constant across SLS prints using polyamide 12 material to isolate the anisotropic effects of orientation and thickness. An evaluation of ultimate tensile strength, modulus of elasticity, strain at failure, yield strength, and Poisson’s ratio, and scanning electron microscope fractography are conducted. Transverse strain and Poisson’s ratio are a key aspect that provide insight into the feasibility of building numerical orthotropic models. These data are used to calculate the degree of anisotropy due to both thickness and orientation. The results support the future use of SLS printing and modeling of thin-walled structures, such as scaled structural ship modeling. The presented data provide guidance on the impact of print orientation and thickness that will aid in manufacturing structural parts with intentionally tuned material properties.

## 1. Introduction

**Figure 1:**
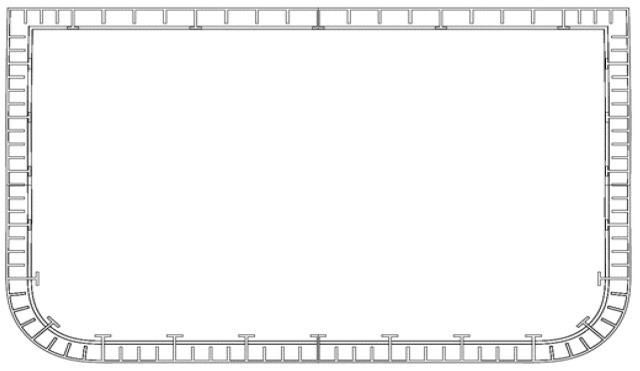
Typical cross-section of a ship model with simplified structural arrangements [3].

**Figure 2:**
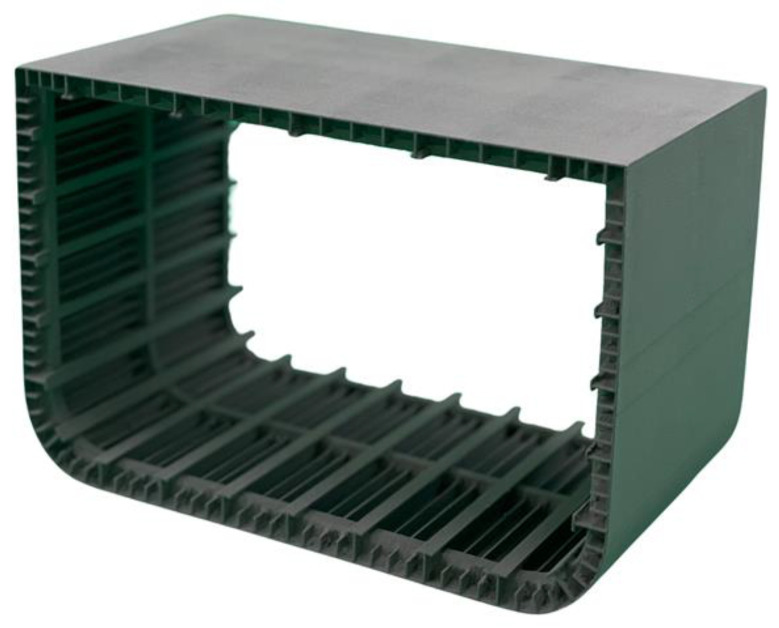
Section of a ship model fabricated using AM [3].

Additive manufacturing (AM) continues to emerge and progress in many fields as a powerful tool to meet a multitude of applications [1,2]. The research performed in this study can be applied to numerous industries and applications and specifically supports investigating the use of AM to fabricate scaled structural ship models for hydro-elastic model testing [3]. Typically, hydro-elastic models are used to evaluate design loads for ships [4,5]. The model’s structural stiffness in hydro-elastic model testing needs to meet the scaled structural stiffness of the full-scale ship [3,6]. The scaling is performed in accordance with Equation (1).

(1) $$I_{model}=\frac{I_{ship}\times E_{ship}}{\lambda^{5}\times E_{model}}$$

where *I_model_* is the area moment of inertia for the beam, *I_ship_* is the area moment of inertia for the full-scale ship cross-section around the axis of interest, *E* is the material Young’s modulus of elasticity for the model or the full-scale ship, and *λ* is the model scale factor. Because of the stiffness scaling, the scaled models need to have relatively large overall dimensions and very narrow shell thickness. An example of these dimensions would be for a model with overall dimensions (in one or more directions) of 200 mm and a shell thickness of less than 1 mm. Additionally, these models include small internal structural details with relatively small dimensions and thicknesses. Figure 1 shows a typical simplified cross-section of a model with small structural details. The requirements for a model with very thin shell thickness and small structural details are not practically achievable using traditional manufacturing methods at a reasonable cost. However, advancements in AM technology in recent years have opened the way to practically fabricating these models with reasonable resources [3]. Figure 2 shows the AM fabricated ship model for the same cross-section shown in Figure 1.

To achieve the desired accurate structural and material properties of the AM fabricated model, a thorough investigation of printed material properties was warranted. Powder bed fusion (PBF) is one of the most prolific methods used to manufacture end use parts [7,8]. Within PBF, Selective Laser Melting (SLM) is a common method to fuse metal and Selective Laser Sintering (SLS) is used to fuse (sinter) polymers. Material property data based on thickness and orientation could allow the tuning of printed PBF parts.

Polyamide 12 (PA12) was selected for this research as it remains one of the most prolific materials used for SLS parts [8,9,10]. PA12 continues to be widely used for SLS due to its relatively low cost, powder reuse ratio, low water absorption, and large sintering window [11,12]. A typical layer height for SLS with neat PA12 is 0.1 mm, which results in minimum wall thicknesses typically around 0.5–0.8 mm [13,14]. Jaksch et al. [14], Sindinger et al. [13], and Tasch et al. [15] found that SLS PA12 tensile properties such as modulus of elasticity, ultimate tensile strength, and yield at break can vary dramatically between thin wall (<1 mm) and standard thicknesses (~2–4 mm). Jaksch’s and Sindinger’s teams also found that print orientation can compound the anisotropy of thin-walled parts manufactured via SLS. These sources of anisotropy can also combine with material property variations due to laser energy density (power, speed, and pattern), temperatures (ambient, top layer, and build plate), and build orientation [16,17]. All these factors must be considered when designing experiments to test individual effects.

A numerical model for a thin wall structure, such as a ship model, can aid in predicting full scale structural performance. For a typical steel ship modeling, the material is assumed to be isotropic and a single material property can be used in all directions. However, isotropy for AM parts cannot be assumed [13,14]. Fully anisotropic models may be required for numerical simulation; however, orthotropic anisotropy modeling warrants investigation based on the substantial simplification of creating, verifying, and using such a model. The characterization and calculation of the degree of anisotropy using techniques established by Alcisto [18] can help quantify the necessity of anisotropic numerical models. It is hypothesized that orthotropic modeling may be a useful method of modeling structures such as a ship model that have most structural elements normal to one another (Figure 1 and Figure 2). To properly scale the stiffness of a ship model, the wall thickness must be relatively thin; and it is also hypothesized that an SLS orthotropic model will depend on the thickness of the structural components. A numerical model for a thin wall structure, such as a ship model, requires all tensile properties including Poisson’s ratio. However, a literature review of published work revealed a lack of Poisson’s ratio data for 3D printed material in general. This research is focused on the two hypotheses discussed above and the feasibility of gathering orthotopic material property data including Poisson’s ratio to be used in thin wall PA12 SLS orthotropic models.

Following the Introduction section in this paper, the Materials and Methods section covers the design of the experiments, including the parameters for the specimens and the equipment used for printing and testing. The next section is Results, which covers all the data collected from tensile testing and fractography results as well as the methods used to analyze the data. A separate Discussion section follows to present the implications of the results. Finally, a Conclusion section covers the conclusions based on the data and the discussion presented in the prior sections.

## 2. Materials and Methods

### 2.1. Design of Experiments

**Figure 3:**
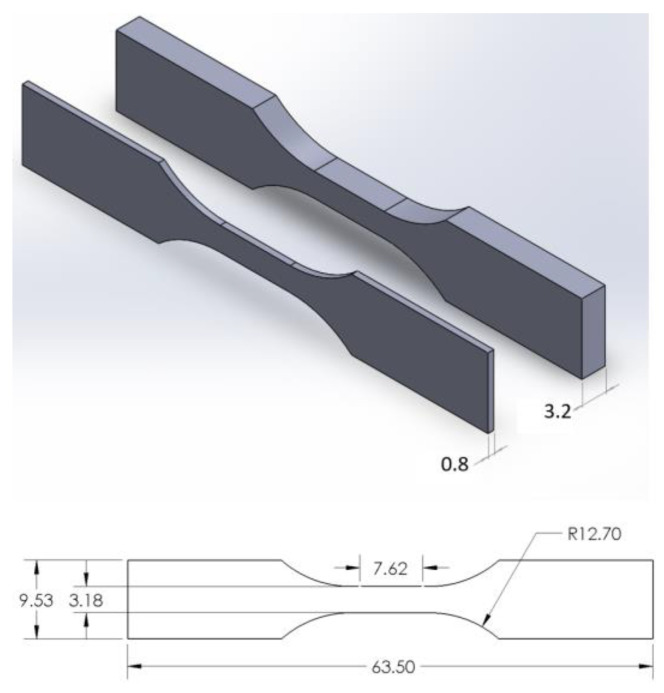
ASTM-D638 Type V tensile specimen dimensions (mm) for both standard (3.2 mm thickness) and thin (0.8 mm thickness) specimens.

**Figure 4:**
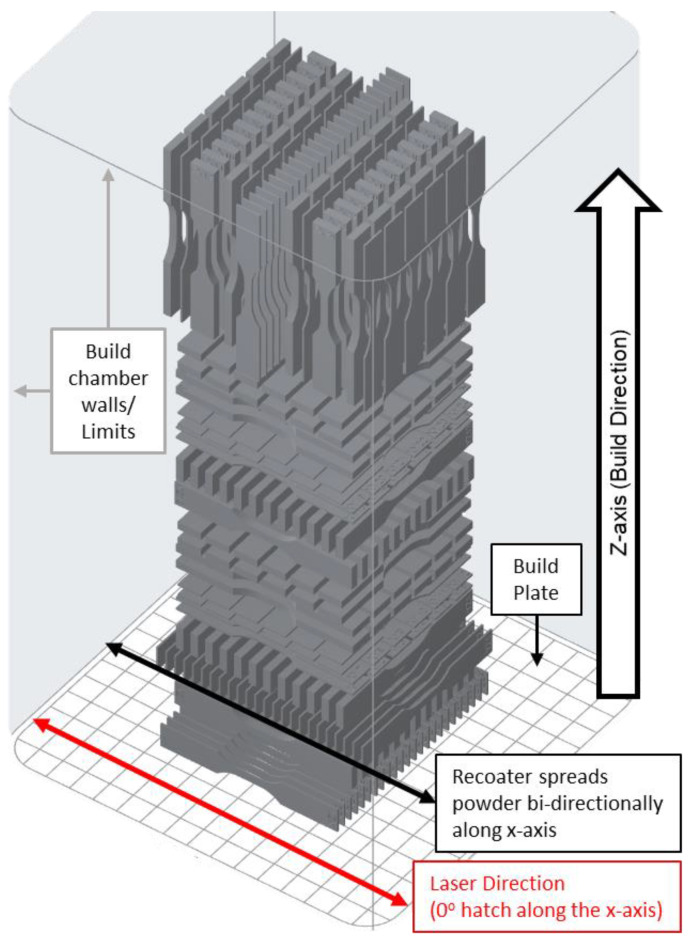
D638 Type V tensile specimen build area distribution.

**Figure 5:**
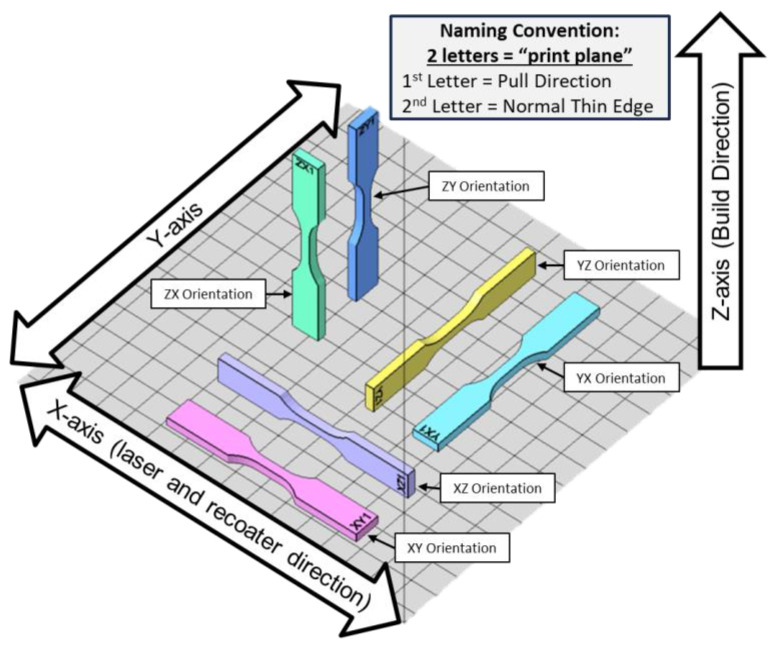
Print orientation and naming convention (Note: Specimen spacing is expanded for clarity. See Figure 4 for printed specimen spacing on build plate).

A design of experiments (DOEs) was developed to analyze thick and thin SLS orthotropic tensile characteristics. ASTM-D638 tensile specimens were selected based on polymer tensile characterization and the ability to manufacture large number of specimens in a single build. In total, 144 specimens were manufactured with half of the specimens printed at a standard 3.2 mm thickness and half printed at a 0.8 mm thickness (Figure 3). This resulted in each orthotropic orientation having 12 standard thickness and 12 thin specimens. All specimens were manufactured in a single print (Figure 4) to minimize slight differences between prints due to the recycled powder thermal history and ambient factors. Some research has also found noticeable differences in tensile properties based on build plate location [15,19] and so specimens were placed throughout the middle 60% of the build area to minimize the print area edge effects and anisotropy due to the build location (Figure 4). Lastly, a naming convention was created to easily identify the specimens based on the print and tensile load orientation. The specimen identifier consists of two letters where the first letter is the pull direction with respect to the print plane and the second letter is the normal direction of the specimen’s long thin edge with respect to the print plane (Figure 5)

### 2.2. Sample Manufacturing

A Formlabs (Formlabs, Somerville, MA, USA) Fuse 1 SLS printer with Formlabs neat PA12 powder was used to print all the specimens. Autodesk (Autodesk, San Francisco, CA, USA) Netfabb AM software(version 2024) tool was used to arrange and orientate all the specimens within the build area and Formlabs PreForm (version 3.29) slicing software was used to slice all the 3D models. Changes in energy density based on the hatch pattern and laser power can affect the mechanical properties of SLS parts [20] and so a constant recoater direction, layer height, hatch pattern, laser speed, laser power, and temperature profile were used throughout the print to isolate changes based on specimen orientation and thickness.

Formlabs PA12 powder [21] is specifically developed for the Fuse Series printers and most powder properties remain proprietary. Mixing unsintered powder from previous SLS prints (recycled powder) with fresh unused powder is a common practice in SLS printing to decrease material waste throughout the manufacturing process. The percentage of fresh powder to total powder is called the refresh ratio. Refresh ratios of 15–50% are common when testing the mechanical properties of SLS prints [22,23,24]. However, the refresh ratio will affect the material crystal structure, powder coalescence, and part porosity and must be considered when comparing parts and material properties [25]. Recycled powder with a 30% refresh ratio was used in this study as this is the manufacture’s recommended ratio when printing with the Fuse 1 [26,27].

**Figure 6:**
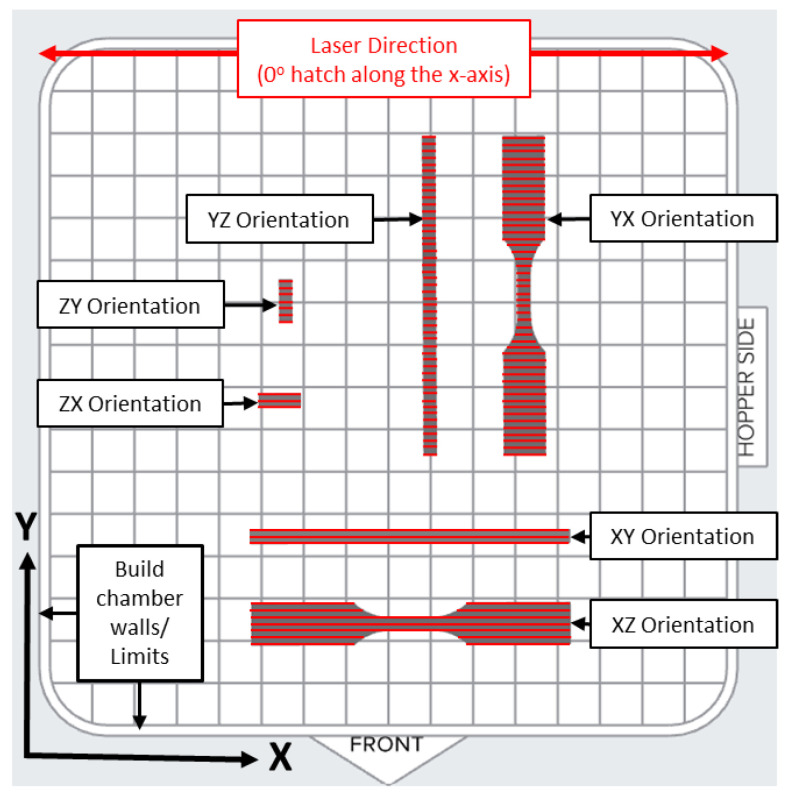
D638 Type V tensile specimen hatch pattern indicated in red for each orientation. Hatch pattern remained constant for each layer (Note: Details in the figure are expanded to show hatch directions).

The Fuse 1 operates with an air chamber environment and internal chamber temperature of up to 200 °C with a 10 W laser (200 microns spot size) (Formlabs, 2024). PreForm auto sets the hatch spacing and laser power and uses a patent-pending “Surface Armor” technology to adjust the scan speed based on a cross-section slice to maintain consistent mechanical properties. To achieve a standard comparison of print orientations, this study used a standard x direction hatch pattern without perimeters (Figure 6) and a 0.110 mm layer height for all the prints. The hatch pattern in Figure 6 is expanded to show the orientation and shape and is not representative of the spacing or total number of laser passes. The specimen spacing in Figure 6 is also expanded to show the hatch detail of each specimen and is not representative of the actual print layout. See Figure 4 for the specimen spacing on the build plate.

### 2.3. Tensile Tests

D638-22 Type V uniaxial tensile test standards were used for all the tensile test procedures with a crosshead rate of 1 mm/sec. The specimens were placed in a 73.4 ± 3.6 °F; 50 ± 10% R.H. laboratory environment for a minimum of 40 h and measured for width and thickness at three distinct cross-sectional planes prior to test initiation in accordance with Procedure A of ASTM D618.

Instron (Instron, Norwood, MA, USA) Model 4466 screw-driven test frame and a Model 2518-806 load cell possessing a verified tensile range of 1.00 to 200lbf provided the means to apply and resolve force, respectively. Each specimen grip section was reinforced with one layer of 120 grit Norton Metalite (Saint-Gobain Industries, Staverton, UK) abrasive paper prior to installation. Additionally, each specimen gauge section was painted to facilitate the acquisition of longitudinal deformation measurements through digital image correlation. The specimen was subsequently centered and restrained within serrated parallel face grips at a pressure sufficient to prevent slippage during force application. Once mounted, a transverse deformation measurement device was centered and frictionally retained across the width of the gauge section. Longitudinal deformation measurements were acquired with a non-contacting digital imaging correlation system consisting of an Imetrum (Imetrum Limited, Bristol, UK) Model SC-2 processor and an Allied Vision (Allied Vision, Thuringia, Germany) Mako Model G-192B PoE camera. Transverse deformation was acquired with an MTS (Illinois Tool Works, Glenview, IL, USA) Model 632.18B-20 measurement device possessing a variable gauge length. Digital data were acquired at a target frequency of 10 Hz. with Imetrum Video Gauge Version 5.4.8 (Build 6128) software.

### 2.4. Data Collection and Reporting

While 12 specimens at each orientation and thickness were printed, load, axial displacement, and transverse displacement were collected for a minimum of three specimens of each orientation and thickness. Additional specimens were used as needed. The following values were analyzed: elastic modulus (E_A_), 0.2% yield strength (σ_y_), ultimate tensile strength (UTS), strain at failure (ε_f_), Poisson’s ratio (ν), and degree of anisotropy (DoA). The standard deviation was calculated based on the population using Equation (2) and reported on the material property figures as error bars.

(2) $$s=\sqrt{\frac{\sum {\left(x-\bar{x}\right)}^{2}}{n-1}}$$

where x represents the individual calculated values of each specimen, $\bar{x}$ is the mean of the values, and n is the number samples.

### 2.5. Fracture Characterization

Microscopy of the fractured tensile surfaces was conducted to further understand the influence of thickness and orientation on the SLS parts. A TESCAN (Brno, Czech Republic) MIRA 3 Scanning Electron Microscope (SEM) was used to capture all the images. The PA12 fracture surfaces were sputter coated with a thin (~5 nm) coating of gold to support a conductive pathway. All the SEM images were obtained while operating at 5 keV in the secondary electron mode and used to analyze the surface morphology, particle coalescence, voids, and edge effects.

## 3. Results

### 3.1. Tensile Test Results

**Figure 7:**
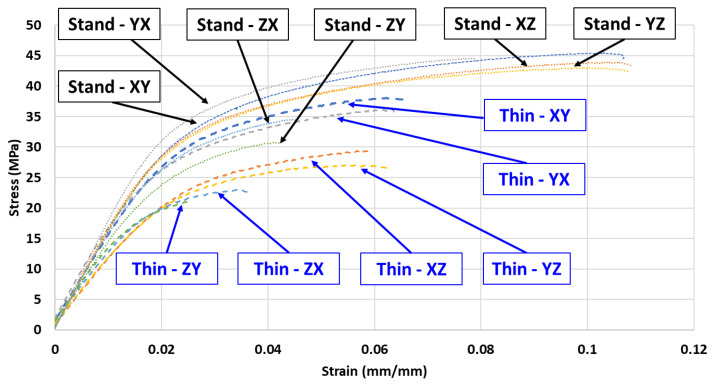
Representative stress–strain curve for each thickness and all six print orientations used in this study showing the noticeable performance differences, particularly for print orientations of ZX and ZY.

The mechanical testing performed on the tensile specimens supported the determination of five key material properties including E_A_, σ_y_, UTS, ε_f_, and ν. These material properties were calculated for each print orientation and thickness and used in Section 3.2 to analyze the degree of anisotropy in the printed samples. A representative stress–strain curve for each thickness and orientation is presented in Figure 7. Material property graphs comparing the effects of thickness and orientation as well as a comparison of the degree of anisotropy for different orientations and thickness are presented and discussed in the following sections.

#### 3.1.1. Axial Elastic Modulus (E_A_)

**Figure 8:**
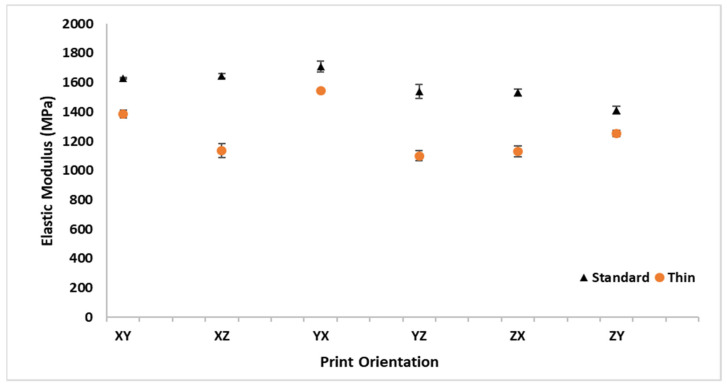
Average axial elastic modulus (E_A_) with standard deviation bars for standard and thin specimens at all orthotropic print orientations.

The E_A_ results are listed in Table 1 and graphically depicted in Figure 8 for all the print orientations and sample thicknesses. The standard thickness samples were consistently stiffer than the thin samples for all the build orientations. The average E_A_ for all the thin samples across all the orientations of 1258.3 MPa was 319.5 MPa (20%) lower than the average standard thickness E_A_ of 1577.8 MPa. The standard thickness YX orientation had the highest average E_A_ value, while the thin YZ sample had the lowest E_A_ value. The YX orientation had the minimum difference in E_A_ between the standard and thin samples (9% decrease in E_A_) and the XZ orientation had the maximum difference between the standard and thin samples (31% decrease in E_A_). A comparison of build orientation performance in Figure 8 shows that E_A_ does not tend to be as consistently influenced by build orientation as by thickness. However, the thin specimens had a noticeable increase in E_A_ for the XY and YX orientations.

#### 3.1.2. Yield Strength (σ_y_)

**Figure 9:**
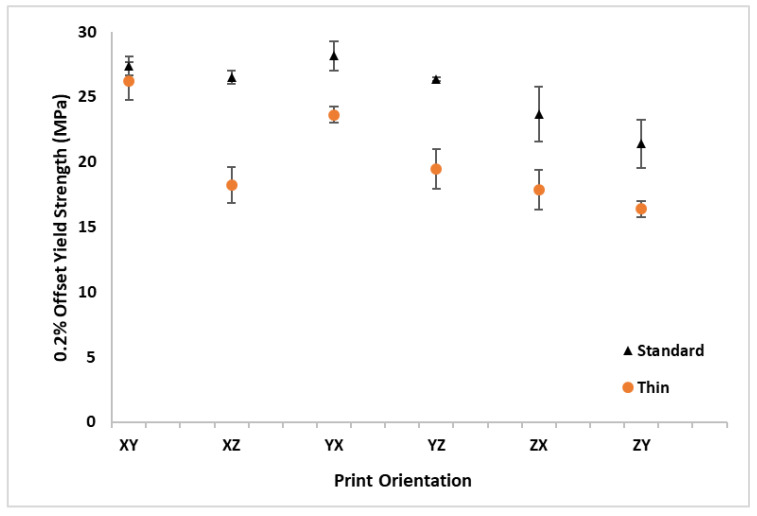
Average 0.2% yield strength (σ_y_) with standard deviation bars for standard and thin specimens at all orthotropic print orientations.

The σ_y_ results are listed in Table 2 and graphically depicted in Figure 9 for all the print orientations and sample thicknesses. A 0.2% strain offset from the E_A_ was used to define σ_y_. Similarly to E_A_, the standard thickness samples had a higher *σ_y_* for all the build orientations. The average σ_y_ for all the thin samples across all the orientations of 20.3 MPa was 5.3 MPa (21%) lower than the average standard thickness σ_y_ of 25.6 MPa. The standard thickness YX orientation had the highest average σ_y_ value, while the thin ZY orientation had the lowest average σ_y_. The XY orientation had the minimum difference in σ_y_ between the standard and thin samples (4% drop in σ_y_ from standard to thin) and the XZ orientation had the maximum difference with a 36% drop in σ_y_ from standard to thin samples. All the specimens with a Z pull orientation had a lower *σ_y_* than the specimens that were printed flat on the build plate.

#### 3.1.3. Ultimate Tensile Strength (UTS)

**Figure 10:**
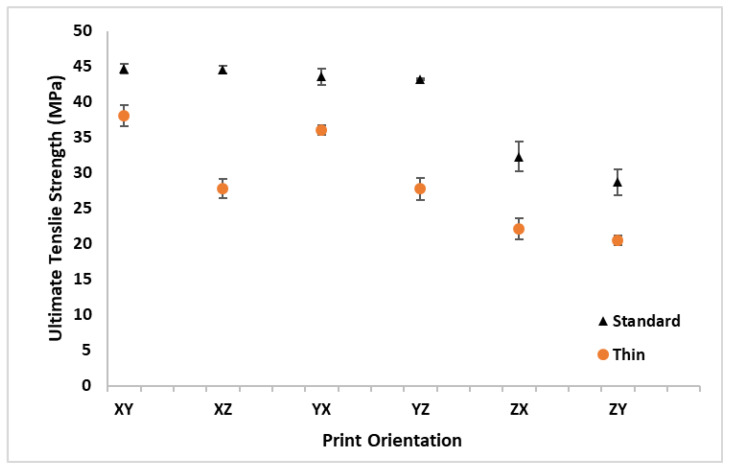
Average ultimate tensile strength (UTS) with standard deviation bars for standard and thin specimens at all orthotropic print orientations.

The UTS results are listed in Table 3 and graphically depicted in Figure 10 for all the print orientations and sample thicknesses. The standard thickness samples were consistently stronger than the thin samples for all the build orientations. The average UTS for all the thin samples across all the orientations of 28.7 MPa was 10.8 MPa (27%) lower than the average standard thickness UTS of 39.5 MPa. The XY orientation had the minimum difference in UTS between the standard and thin samples (15% drop in UTS from standard to thin) while the XZ orientation had the maximum difference with a 38% drop in UTS from the standard to thin samples. A comparison of the build orientation performance in Figure 10 indicates similar trends to σ_y_ (Figure 9). Like σ_y_*,* the thin specimens had a noticeable increase in UTS for the XY and YX orientations. The standard thickness XY orientation had the highest average UTS value, which was just slightly above the XZ, YX, and YZ orientations. Also, like σ_y_*,* all the specimens with a Z pull orientation had a lower UTS than the specimens that were printed flat on the build plate.

#### 3.1.4. Strain at Failure (ε_f_)

**Figure 11:**
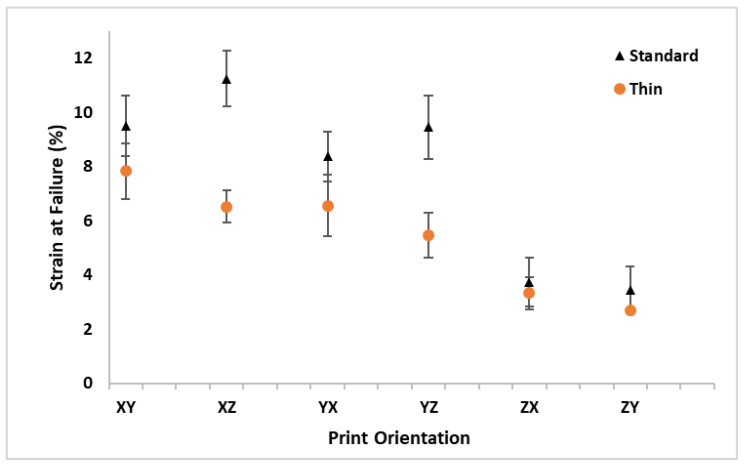
Average strain at failure (ε_f_) with standard deviation bars for standard and thin specimens at all orthotropic print orientations.

The ε_f_results are listed in Table 4 and graphically depicted in Figure 11 for all the print orientations and sample thicknesses. The standard samples typically had a higher ε_f_ than the thin samples; however, there was a minimal difference with the standard deviation overlap for the vertically printed specimens (ZX and ZY). The average ε_f_ for all the thin samples across all the orientations of 5.4% was 29% lower than the average standard ε_f_ of 7.6%. The standard thickness XZ orientation had the highest average ε*_f_* while the thin ZY samples had the lowest average ε_f_ value. The ZX orientation had the minimum difference in ε_f_ between the standard and thin samples (11% drop in ε_f_ from standard to thin) while the YZ orientation had the maximum difference with a 42% drop in ε_f_ from the standard to thin samples. A comparison of build orientation performance in Figure 11 shows the standard thickness samples had a higher ε_f_ for the samples printed along the print bed. However, in contrast to the other properties, for the samples printed along the *z*-axis, the standard and thin samples failed at nearly the same strain. Additionally, unlike the previous properties, the thin specimens did not have a noticeable increase in ε_f_ for the XY and YX orientations.

#### 3.1.5. Poisson’s Ratio (ν)

**Figure 12:**
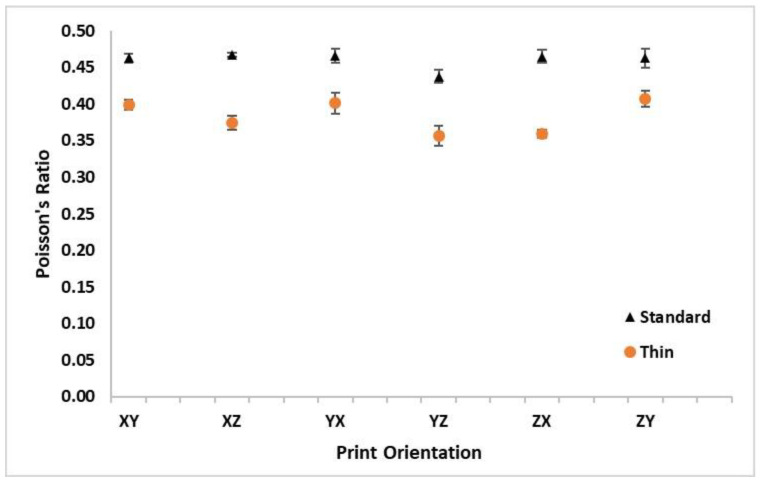
Average Poisson’s ratio (ν) with standard deviation bars for standard and thin specimens at all orthotropic print orientations.

The ν results are listed in Table 5 and graphically depicted in Figure 12 for all the print orientations and sample thicknesses. The standard thickness samples consistently had a higher ν than the thin samples for all the build orientations. The average ν for all the thin samples across all the orientations of 0.383 was 17% lower than the average standard ν of 0.460. The standard thickness XZ orientation had the highest average ν value; however, this value was very similar to all the other orientations for the standard thicknesses, other than the YX orientation, which had a slightly lower average ν value. In contrast to the previous properties, the samples printed in the z-direction (ZX and ZY) had both the minimum and maximum difference in ν between the standard and thin samples. The ZY orientation had the minimum difference in ν between the standard and thin samples (12% drop in ν from standard to thin samples) while the ZX orientation had the maximum difference with a 23% drop in ν from the standard to thin samples. The overall trends shown in Figure 12 show very little change in ν based on orientation when compared to the other material properties analyzed. This is particularly true for the specimens printed along the *z*-axis (ZX and ZY).

### 3.2. Degree of Anisotropy Analysis (D_o_A)

The D_o_A was analyzed for all five key material properties (E_A_, σ_y_, UTS, ε_f_, and ν) that were evaluated during the mechanical testing. The influence of both build orientation and part thickness on material properties was calculated using Equation (3) and Equation (4), respectively:

(3) $$D_{O}A_{\mathrm{orientation}12}=\frac{{\bar{M\_\mathrm{orientation}}}_{1}-{\bar{M\_\mathrm{orientation}}}_{2}}{{\bar{M\_\mathrm{orientation}}}_{\mathrm{avg}}}\times 100\%$$

(4) $$D_{O}A_{\mathrm{thickness}12}=\frac{{\bar{M\_\mathrm{thickness}}}_{1}-{\bar{M\_\mathrm{thickness}}}_{2}}{{\bar{M\_\mathrm{thickness}}}_{\mathrm{avg}}}\times 100\%$$

where D_o_A_orientation12_ represents the degree of anisotropy at two different orientations (orientation “1” and “2”). D_o_A_thickness12_ represents the degree of anisotropy at two different thicknesses (thickness “1” and “2”). A similar method was used by Alcisto and Sindinger ([13,18]) to describe the degree of anisotropy; however, in Equations (3) and (4), M_orientation_avg_ and M_thickness_avg_ is the average of the two material properties at the two different orientations (M_orientation_avg_) or thicknesses (M_thickness_avg_). The numerator values of Equation (3) compare the anisotropic effects of orientation at a specific thickness; while the numerator values of Equation (4) compare the anisotropic effects of thickness at a specific orientation. Holding one design variable constant improves the isolation of the impact of either the print orientation or part thickness on the material properties.

The degree of anisotropy values are presented in Table 6 for all the print orientations and thicknesses. The degree of anisotropy based on orientation (D_o_A_orientation_) was analyzed by comparing the anisotropy between all the print orientations with thickness fixed. The minimum, maximum, and average D_o_A_orientation_ are presented for both the standard and thin specimens. Each property has orientations with a minimum D_o_A_orientation_ near zero (negligible anisotropy impacts between these orientation) but also has a maximum D_o_A_orientation_ typically well above zero (notable anisotropy impacts between these orientations). Strain at failure and UTS have notably higher degrees of anisotropy due to the changes in thickness than all the other properties evaluated while ν has the least anisotropy due to thickness. Yield strength and E_A_ have similar average anisotropy due to thickness around 23–24%.

The degree of anisotropy based on thickness (D_o_A_thickness_) was analyzed for each orientation. Overall, ε_f_, thickness has a greater effect on anisotropy than orientation (average D_o_A_thickness_ > average D_o_A_orientation_) for all the material properties evaluated. Additionally, in contrast to the minimum D_o_A_orientation_ being near zero for all the material properties, the minimum DoA_thickness_ is 4.3% for σ_y_ and 10–16% for all the other properties. Like anisotropy due to orientation, ε_f_ and UTS have the most anisotropy due to thickness while Poisson’s ratio has the least anisotropy. However, the maximum D_o_A_orientation_ is much higher than the maximum DoA_thickness_ for ε_f_. It is also notable that the build direction (*z*-axis) has the most influence on thickness-based anisotropy for E_A_, σ_y_*,* and UTS, while printing along the XY plane (flat on the build plate) minimizes anisotropy due to changes in thickness.

### 3.3. Fractography Results

**Figure 13:**
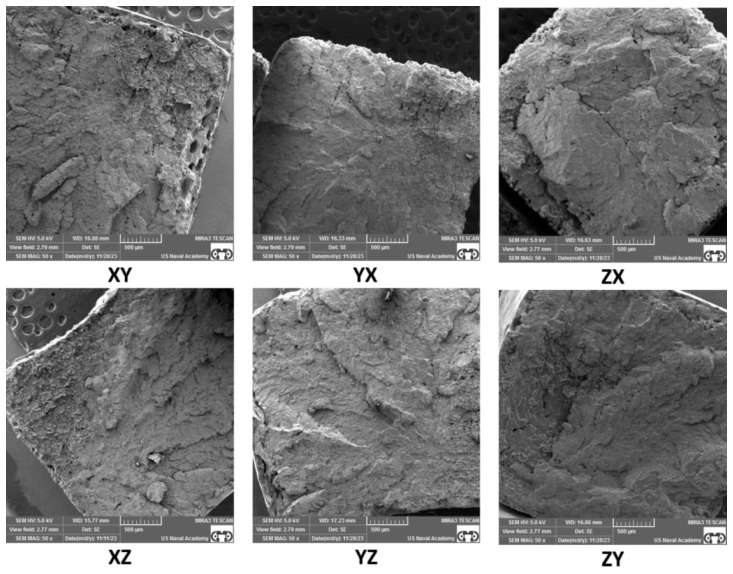
Standard thickness specimen SEM fractography at 50× magnification.

**Figure 14:**
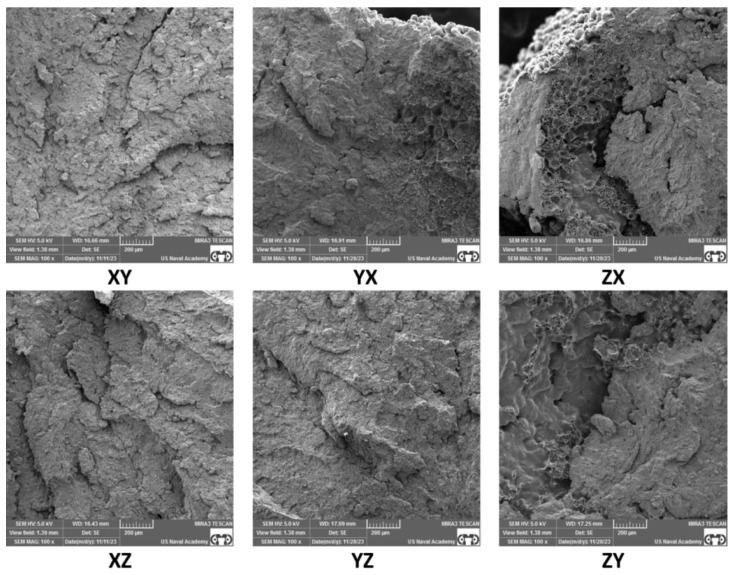
Standard thickness specimen SEM fractography at 100× magnification.

**Figure 15:**
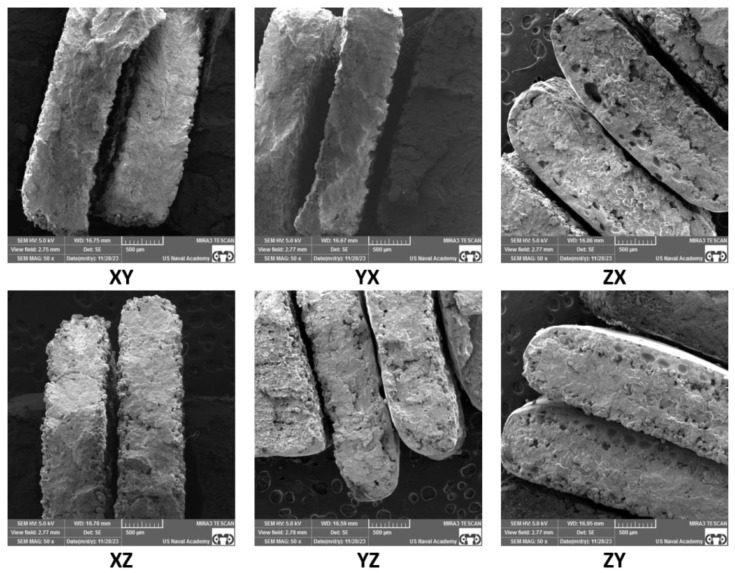
Thin thickness specimen SEM fractography at 50× magnification.

**Figure 16:**
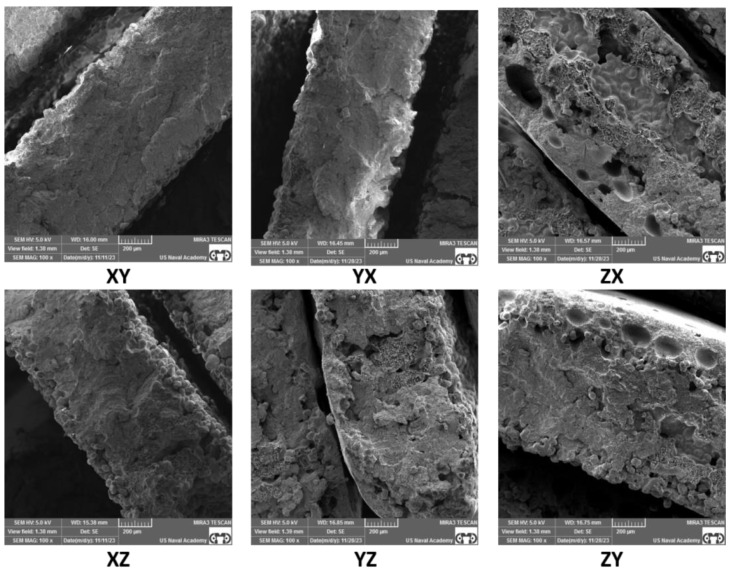
Thin thickness specimen SEM fractography at 100× magnification.

SEM of the tensile specimen fracture morphology was conducted to further understand the factors that may affect anisotropy in SLS samples. A representative fracture image of each build orientation and each sample thickness is presented in Figure 13, Figure 14, Figure 15 and Figure 16. The fractography images for the standard thickness samples are presented in Figure 13 at 50× magnification and in Figure 14 at 100× magnification. The fractography images for the thin samples are presented in Figure 15 at 50× magnification and in Figure 16 at 100× magnification. All the fracture surfaces indicate a relatively brittle fracture with minimal plastic deformation or necking. These data match the quantitative strain data with less than 0.5% strain from UTS to failure for all the specimens except the XZ orientation for the standard specimens which had an average strain of 0.7% from UTS to failure. All the SEM images of the fracture surfaces were evaluated to gain an understanding of the impact of porosity, voids, and inadequate sintering/particle coalescence on the material properties.

In general, the thin samples had more voids and porosity than the standard samples. This can be clearly seen in Figure 16-YZ, ZX, and ZY, which show appreciably more voids than the same orientations for the standard samples shown in Figure 14. The presence of voids and porosity can certainly impact material properties, to include strength and elastic modulus, which is discussed by Al-Maharma et al. [28]. The fractography analysis of the thin samples shows a greater edge effect than the standard samples. There appears to be inadequate sintering on the outer portions of most of the samples, but this effect is more pronounced on the thin samples. This inadequate sintering at the edges is most clearly seen in Figure 16 and presents as nearly spherical particles without clear bonding to near neighbor particles and the lack of a smooth edge surface. The sintering issues can be attributed to inadequate heat supply during the build process due in part to the short hatches compared to the standard sample hatch. Further testing and evaluation of this phenomenon, to include mitigation techniques, is warranted.

For all the samples, voids and porosity are much more prevalent for the samples printed in the build direction (ZX and ZY orientations in Figure 14 and Figure 16). While porosity was not quantitatively evaluated in this study, the presence of pores and voids is likely the result of build orientation challenges related to energy density with the short laser segments (hatch) and tensile load being applied along the layer-to-layer bonding as opposed to being applied along the layers [27,28,29],[30]. Additionally, while some of the samples, particularly the standard samples printed in the XY, XZ, and YZ direction and to an extent the thin samples printed in the XY, XZ, and YZ direction, exhibit a fairly homogenous microstructure, the remainder of the samples show a more heterogenous structure with substantial differences that could be responsible for impacting the material properties. Finally, it is relevant to note that for all the samples printed in the X and Y directions, the build layer delineation characteristics often seen in SLS PA12 prints [31] are not present, indicating that the layer-to-layer sintering appears to be adequate with the prescribed print parameters for samples printed along the bed. Overall, the SEM fractography showed differences in particle coalescence, void spacing, porosity, and sintering, with differences based on thickness and orientation. Ultimately, these differences impacted the load-bearing cross-section area and give insight into the anisotropic material property performance.

## 4. Discussion

This study reasonably isolated and analyzed two anisotropic drivers: thickness and print orientation. The standard deviation for all the sets of samples was less than 10% for all the reported properties at each orientation and thickness except for strain at failure. This indicates that while some variability may exist based on the location of the sample in the build area during the print, overall, the DOE did mitigate the impact of anisotropy due to the build location. Thus, the variability of the material properties is mostly due to the print orientation or thickness. The order of sensitivity of the material properties to thickness from most sensitive to least sensitive is as follows: UTS, ε_f_, σ_y_, E_A_, and ν. The order of sensitivity of the material properties to print orientation from the most sensitive to the least sensitive is ε_f_, UTS, σ_y_, E_A_, and ν. UTS and ε_f_, are the only two properties that do not have the same sensitivity order between the thickness and print orientation. However, the sensitivity of UTS and ε_f_, to thickness is nearly the same (within 1.5%). It is interesting to note that anisotropy due to both orientation and thickness seems to be more apparent for the material properties well within the plastic region (UTS and ε_f_).

### 4.1. Sensitivity of Properties to Thickness (Thickness Anisotropy)

The standard specimens had increased material properties values compared to the thin specimens across all the material properties evaluated, which is clearly supported by the fractography images (Figure 13, Figure 14, Figure 15 and Figure 16). The thin sample images (Figure 15 and Figure 16) not only show an increased amount of internal porosity, but also a large amount of incomplete particle sintering along the edges. These data coincide with the findings of Al-Maharma et al. [28] and with ε*_f_* quantitative data (average thin ε*_f_* 29% less than average ε*_f_* for standard samples). The under-sintered edge effects observed in the SEM fractography images are not only dimensionally larger for the thin specimens, but also appear to encompass a much higher percentage of the cross-sectional area. This greatly influences the strength and stiffness of the material. This edge effect was particularly noticeable between the standard (Figure 14) and thin (Figure 16) specimens in the XZ and YZ orientation, which helps explain the drastic decrease in E_A_, σ_y_, UTS, and ε_f_, for these two orientations. This sintering difference contributed to the thin specimens being more brittle than the standard specimens (Figure 7), which led to the large DoA_thickness_ for these properties. A constant scan pattern for all the thicknesses appears to isolate the effects due to thickness when compared to Rodriguez et al. [16] and Sindinger et al. [13], who varied the laser and hatch settings based on the specimen thickness. This comparison also highlights the benefit of customizing laser pattern and energy density to mitigate thickness anisotropy, which coincides with the results of Jaksch et al. [14].

Interestingly, in contrast to XZ and YZ, strain at failure had less difference for the vertical prints (ZX and ZY orientations) between the standard and thin specimens. Both the standard and thin samples exhibit a relatively brittle fracture when printed vertically (Figure 7 and Figure 11), which led to the lower maximum ε_f_ DoA_thickness_. This is likely due to the difference in sintering between the layers as opposed to between or along the laser movement (hatch) on a single layer. Han et al. [7] and Hejmady et al. [32] noted similar findings and this warrants potential future work to investigate post-sintering heat treatments to fully fuse the layers. Heat-treating metal powder bed fusion parts has been relatively widely studied [33,34,35]. However, the full benefits of heat-treating polymer-based powder bed fusion parts is relatively under-investigated [36]. Based on the thin specimen results of the present study, post-sintering treatments may be beneficial in decreasing thickness anisotropy and warrant future investigation.

### 4.2. Sensitivity of Properties to Print Orientation (Print Orientation Anisotropy)

The vertically printed specimens (ZX and ZY orientations) exhibited a decrease in values across all the material properties except ν, which had relatively constant values for all orientations (DoA_max_standard_ = 6.5% and DoA_max_thin_ = 13.3%). Poisson’s ratio is a parameter that is not often evaluated but critical to building anisotropic numerical models. These sensitivities and data are crucial to analyzing the importance of building an orthotropic model as opposed to an isotropic model for applications such as scaled structural ship models [3]. If there is not a significant D_o_A_orientation_, it is likely beneficial to use a more simplistic isotropic model. However, if D_o_A_orientation_ is large, an anisotropic model is likely required to accurately predict the model and full-scale performance. Uniquely to this study, ν appears to be nearly independent of the print orientation when the hatch pattern is held constant across all the orientations. Poisson’s ratio is the only material property studied that is simultaneously influenced by strain in two orthotropic orientations (axial and transverse strain). Future work should be done to investigate if this is due to the simultaneous interactions of the axial and transverse anisotropic strains, which may essentially mitigate the overall orthotropic effects, ν. It is also important to note that it appears this is also influenced by ν being measured well within the elastic range of the material as opposed to the other properties that were measured well within the plastic range of the material. This should also be studied with E_A_ in mind which was also less impacted by the print orientation. However, E_A_ could also potentially be influenced by the fact that it is simultaneously affected by both stress and strain, the key difference between E_A_ and ν being that the factors affecting E_A_ act in the same axial direction while the factors affecting ν are affected by D_o_A_orientation_.

Strain at failure was the most influenced by orientation with a drastic decrease in ε_f_ for both the standard and thin vertical prints (~3.5% for standard and 3.0% for thin specimen). This is consistent with other literature and can be clearly seen in the fractography images [7,32]. As seen in Figure 16, at 100× magnification, there is more visible internal porosity and slightly less effective sintering in the standard ZX and ZY samples as compared to all the other print orientations. Additionally, similar trends are seen for the thin samples with substantially more internal porosity and poor sintering for the ZX and ZY print orientations. While the edge effects, showing a lack of sintering at the sample edges for the other orientations could impact strain at failure, the large number of internal pores and voids likely have a more substantial impact. Interestingly, ε_f_ is also the only property that had a higher D_o_A_orientation_ for the standard specimens than the thin specimens. However, as discussed earlier, it is also important to realize that ε_f_ is also the property analyzed with the most plastic deformation and thus this could have been due to simply having the most variance given the number of tested specimens. We recommend further testing with a large number of specimens if this relationship is critical to a particular part application.

The degree of anisotropy based on print orientation for UTS and *σ_y_* was greater than the D_o_A_orientation_ for ν and E_A_, but less than the D_o_A_orientation_ for ε_f_. The UTS and *σ_y_* values for the two Z print orientations (ZX and ZY) were the lowest and most impacted by orientation (average vertical print UTS_standard_ = 30.5 MPa, UTS_thin_ = 21.3 MPa, σ_y_standard_ *=* 22.5%, *σ_y_thin_ =* 17.1%). However, the two thin specimen print orientations (XZ and YZ) exhibited substantially lower UTS (~27.8 MPa) and σ_y_ (~18.8 MPa) values as well. As seen in the SEM images presented in Figure 16, the increased porosity and reduced sintering is likely a contributor to these impacts. In general, there was little change due to orientation in the UTS and *σ_y_* values for the XY, XZ, YX, and YZ standard samples, which can be contributed to the relatively consistent sintering throughout these orientations of the standard samples (Figure 13 and Figure 14). Evaluation of the SEM images in Figure 16 for the XZ and YZ thin samples also indicates substantial edge effects of inadequate sintering, which could explain the drop in UTS and yield strength as compared to the XY and YX print orientations. These findings also warrant the investigation of specialized hatch patterns and/or post-processing techniques such as heat treatments and coatings for thin SLS parts.

### 4.3. Combined Sensitivity of Properties to Thickness and Print Orientation (Combined Anisotropy)

All the calculated material properties other than ν are primarily influenced by a value in a single direction and as such there are clear differences in the material properties based on orientation. However, thickness does appear to mitigate the effects of orientation, with the average maximum D_o_A_orientation_ for all properties being 20% for the standard specimens and 25% for the thin specimens. This is particularly true for E_A_, σ_y_ and ν, which have degrees of anisotropy decreases of 49%, 44%, and 67%, respectively, from the thin to the standard specimens. This improvement can be tied to the visibly higher level of porosity in the thin specimens and the poor sintering around the thin sample edges; however, it may also be influenced by the fact that these specimens are predominately influenced by elastic deformation.

## 5. Conclusions

Anisotropy due to orientation and thickness was evaluated by printing all the specimens in a single print with a constant laser setting and scan pattern for all the specimens. Anisotropy was consistently highest for the material properties evaluated while in the plastic region and at a minimum for the material properties well within the elastic region. However, print orientation and part thickness affected all the evaluated properties.

Print thickness affected all the material properties with the thin (0.8 mm) specimens having lower values than the standard (3.2 mm) specimens across all print orientations. The overall order of sensitivity of material properties to thickness, from most to least sensitive, was UTS, ε_f_, σ_y_, E_A_ and ν, with the two highest average DoA_thickness_ being 32.5% (UTS) and 31.0% (ε_f_). These results highlight the importance of referencing material properties based on part thickness to accurately predict the printed part performance. Future research should also investigate the effects of varying hatch patterns and energy density between thick and thin parts to decrease the severity of the drop in material properties from thick to thin part features.

Print orientation impacted the overall material properties, with samples printed along the build/vertical axis (ZX and ZY orientations) being the most negatively impacted by orientation. This impact generally results in the highest sensitivity of anisotropy due to orientation being between the prints in the build direction versus the prints on the build surface (XY, YZ, YX, and YZ orientations). The overall order of sensitivity of the material properties to print orientation was ε_f_, UTS, σ_y_, E_A_ and ν, with a maximum DoA_orientation_ of ε_f_ being 106% for the standard samples and 98% for the thin samples. The identification of the overall sensitivity of these material properties to print orientation provides guidance to optimize SLS print orientation based on end-use requirements.

The present work underlines the importance of tuning energy density (primarily hatch pattern, laser power, and laser speed) based on wall thickness and orientation to maximize mechanical properties. These findings also highlight the possibility of using orthotropic models based on orientation and thickness to predict the performance of thin end-use SLS parts such as scaled structural ship models [3].

Future work should also investigate the post-processing heat treatments of thin-walled prints to reduce edge effects and anisotropy by increasing the overall level of sintering in all directions. The present results show this may be heavily influenced by wall thickness and the fraction of the cross-section that is affected by sintered edge particles. Finally, the ability to blend in additives in the form of fibers or particles is another option to achieve the desired material properties [23]; however; the impacts of these additives on the sintering process and overall print quality may severely degrade thin specimens and will have to be fully characterized to realize the benefits [37]. Overall, this points to the importance of investigating material additives prior to printing, energy density customization during printing, and post-printing strategy after printing to minimize the anisotropy of SLS prints.

## Figures and Tables

**Table 1:** Average axial elastic modulus (E_A_) with standard deviation for standard and thin specimens at all orthotropic print orientations.

Orientation			XY	XZ	YX	YZ	ZX	ZY	Average
**Thickness**	**Standard**	**E_A_ (MPa)**	1629.5	1645.5	1707.6	1539.8	1532.9	1411.1	**1577.8**
		**SD**	4.0	15.9	38.0	45.9	24.2	27.9	
	**Thin**	**E_A_ (MPa)**	1383.5	1135.3	1546.7	1100.9	1130.7	1252.5	**1258.3**
		**SD**	26.1	48.9	8.0	34.7	36.5	19.9	

**Table 2:** Average 0.2% yield strength (σ_y_) with standard deviation for standard and thin specimens at all orthotropic print orientations.

Orientation			XY	XZ	YX	YZ	ZX	ZY	Average
**Thickness**	**Standard**	**σ_y_ (MPa)**	27.4	26.5	28.2	26.3	23.7	21.4	**25.6**
		**SD**	0.6	0.5	1.8	0.4	1.1	0.4	
	**Thin**	**σ_y_ (MPa)**	26.2	18.2	23.6	19.4	17.8	16.4	**20.3**
		**SD**	1.7	1.2	1.4	1.5	1.4	1.0	

**Table 3:** Average ultimate tensile strength (UTS) with standard deviation for standard and thin specimens at all orthotropic print orientations.

Orientation			XY	XZ	YX	YZ	ZX	ZY	Average
**Thickness**	**Standard**	**UTS (MPa)**	44.7	44.6	43.6	43.1	32.3	28.7	**39.5**
		**SD**	0.7	0.5	1.1	0.1	2.1	1.8	
	**Thin**	**UTS (MPa)**	38.1	27.8	36.0	27.8	22.1	20.5	**28.7**
		**SD**	1.5	1.4	0.6	1.5	1.5	0.6	

**Table 4:** Average strain at failure (ε_f_) with standard deviation for standard and thin specimens at all orthotropic print orientations.

Orientation			XY	XZ	YX	YZ	ZX	ZY	Average
**Thickness**	**Standard**	**ε_f_ (%)**	9.5	11.2	8.4	9.4	3.7	3.4	**7.6**
		**SD**	1.1	1.0	0.9	1.2	0.9	0.9	
	**Thin**	**ε_f_ (%)**	7.8	6.5	6.6	5.5	3.3	2.7	**5.4**
		**SD**	1.0	0.6	1.1	0.8	0.6	0.1	

**Table 5:** Average Poisson’s ratio (ν) with standard deviation for standard and thin specimens at all orthotropic print orientations.

Orientation			XY	XZ	YX	YZ	ZX	ZY	Average
**Thickness**	**Standard**	**ν**	0.463	0.467	0.466	0.438	0.465	0.463	**0.460**
		**SD**	0.007	0.003	0.010	0.009	0.009	0.013	
	**Thin**	**ν**	0.399	0.374	0.402	0.357	0.359	0.408	**0.383**
		**SD**	0.007	0.010	0.014	0.014	0.006	0.011	

**Table 6:** Orientation and thickness: degree of anisotropy comparison.

			Axial Elastic Modulus		Yield Strength		Ultimate Tensile Strength		Strain at Failure		Poisson’s Ratio	
			Orient	DoA	Orient	DoA	Orient	DoA	Orient	DoA	Orient	DoA
**Orientation**	**Standard**	**DoA_min**	YZ-ZX	0.4%	XZ-YZ	0.7%	XY-XZ	0.3%	XY-YZ	0.6%	XY-ZY	0.0%
		**DoA_max**	YX-ZY	19.0%	YX-ZY	27.3%	XY-ZY	43.7%	XZ-ZY	106.4%	XZ-YZ	6.5%
		**Average**	8.1%		12.1%		21.1%		55.0%		2.3%	
	**Thin**	**DoA_min**	XZ-ZX	0.4%	XZ-ZX	2.2%	XZ-YZ	0.1%	YX-XZ	0.5%	YZ-ZX	0.7%
		**DoA_max**	YX-YZ	33.7%	XY-YZ	46.2%	XY-ZY	60.1%	XY-ZY	97.8%	YZ-ZY	13.3%
		**Average**	15.9%		21.5%		30.0%		48.1%		7.1%	
**Thickness**		**DoA_min**	YX	9.9%	XY	4.3%	XY	15.9%	ZX	11.2%	ZY	12.6%
		**DoA_max**	XZ	36.7%	XZ	37.1%	XZ	46.4%	YZ	53.5%	ZX	25.7%
		**Average**	23.0%		23.9%		32.5%		31.0%		18.4%	

## Data Availability

Data is contained within the article.
